# 3D Printing Approach in Dentistry: The Future for Personalized Oral Soft Tissue Regeneration

**DOI:** 10.3390/jcm9072238

**Published:** 2020-07-15

**Authors:** Dobrila Nesic, Birgit M. Schaefer, Yue Sun, Nikola Saulacic, Irena Sailer

**Affiliations:** 1 Division of Fixed Prosthodontics and Biomaterials, University Clinic of Dental Medicine, University of Geneva, Rue Michel-Servet 1, CH-1211 Geneva 4, Switzerland; Yue.Sun@unige.ch (Y.S.); irena.sailer@unige.ch (I.S.); 2 Geistlich Pharma AG, Bahnhofstrasse 40, CH-6110 Wolhusen, Switzerland; birgit.schaefer@geistlich.ch; 3 Department of Cranio-Maxillofacial Surgery, Inselspital, Bern University Hospital, University of Bern, Freiburgstrasse 10, CH-3010 Bern, Switzerland; nikola.saulacic@insel.ch

**Keywords:** 3D printing, oral soft tissues, gingiva, biomaterials, tissue engineering, PRF

## Abstract

Three-dimensional (3D) printing technology allows the production of an individualized 3D object based on a material of choice, a specific computer-aided design and precise manufacturing. Developments in digital technology, smart biomaterials and advanced cell culturing, combined with 3D printing, provide promising grounds for patient-tailored treatments. In dentistry, the “digital workflow” comprising intraoral scanning for data acquisition, object design and 3D printing, is already in use for manufacturing of surgical guides, dental models and reconstructions. 3D printing, however, remains un-investigated for oral mucosa/gingiva. This scoping literature review provides an overview of the 3D printing technology and its applications in regenerative medicine to then describe 3D printing in dentistry for the production of surgical guides, educational models and the biological reconstructions of periodontal tissues from laboratory to a clinical case. The biomaterials suitable for oral soft tissues printing are outlined. The current treatments and their limitations for oral soft tissue regeneration are presented, including “off the shelf” products and the blood concentrate (PRF). Finally, tissue engineered gingival equivalents are described as the basis for future 3D-printed oral soft tissue constructs. The existing knowledge exploring different approaches could be applied to produce patient-tailored 3D-printed oral soft tissue graft with an appropriate inner architecture and outer shape, leading to a functional as well as aesthetically satisfying outcome.

## 1. Introduction

Recent years have seen an expansion of the field of three-dimensional (3D) printing, also referred to as additive manufacturing or solid freeform fabrication [1,2]. 3D printing technology allows the production of an individualized 3D object based on a material of choice and a specific computer-aided design. In the medical field, the possibility to include living cells in the procedure has lifted 3D printing to another level and opened a myriad of possibilities for the creation of different tissues. The new opportunities are now paving the way towards patient-tailored treatments. Several factors have contributed to the emerging applications of the 3D printing approaches. The development of a variety of printable biomaterials now offers more precise control of scaffold inner architecture and outer shape. The available analytical digital tools offer quick and precise acquisition and documentation of the patient-specific situation in 3D. An easy transfer of digital data allows the design of anatomically perfectly shaped structures that can be customized for each patient. The expiration of the key 3D printing patents has substantially decreased the cost of printers. The rapid developments of these technologies bring new and exciting approaches in all medical fields, including dentistry. A timeline illustrating the major discoveries of the 3D printing technologies and their applications in medicine is provided in Table 1.

The 3D printing process begins with a design of a 3D model, created by a computer-aided design (CAD) software. The model is then converted into cross-sectional slices and sent to the 3D printer, which deposits layer after layer of the chosen material to produce an object. Such “additive manufacturing” has several advantages over conventional, subtractive manufacturing: (1) it allows the production of a controlled inner structure, (2) it reduces material waste, (3) the object is produced as a single unit instead of being assembled from individual parts, and (4) the designed files can be transferred electronically, easily shared and indefinitely stored without occupying physical space. Consequently, production time and costs are decreased.

In the last decade, 3D printing technology has been broadly used in different medical fields including regenerative medicine, the production of anatomical models and surgical guides as well as for drug formulations [4,5,6,7]. In parallel, the development of 3D printable biomaterials to build tissue models without or with cells enables studying the processes of complex cellular interactions during tissue formation, maturation and disease, as well as toxicology testing and drug screening [4,8,9,10]. 3D-printed models have been used for presurgical planning in craniomaxillofacial surgery [11], cardiology [12], cerebral aneurysm [13] as well as in orthopaedics [14]. Today, physical models are employed as cutting guides for tumor resection as well as templates for shaping patient’s specific implants and prostheses [15,16,17]. Finally, 3D printing has found use in producing anatomical models for education and training [18,19]. This scoping review provides a brief summary of 3D printing approaches in the medicinal field, with a particular emphasis on the current status of 3D printing in dentistry, and the possibilities it offers for personalized soft tissue volume augmentation.

## 2. The 3D Printing Technology

In 3D printing, objects are fabricated automatically by adding material(s) layer-after-layer, to form a 3D volumetric structure [20]. As with any new technology, technical standards had to be established for a wide range of materials, products, systems, and services. The American Section of the International Association for Testing Materials (ASTM) International Standard Organization committee F42 on AM technologies has named seven additive manufacturing categories: binder jetting, direct energy deposition, material extrusion, material jetting, powder bed fusion, sheet lamination, and vat photopolymerization [21]. In the biomedical field, the mainly employed printing methods can be broadly divided into acellular techniques comprising stereolithography (SLA), powder-fusion printing (PFP), solid freeform fabrication (SFF) and techniques including cells: inkjet-based, extrusion-based, and laser-assisted bioprinting (LAB) (reviewed in [3]). SLA is based on beaming a laser or a light source onto a photosensitive polymer to harden its surface. The continuous vertical lifting of the container with a polymer results in a gradual hardening of the material and emergence of a 3D object. SLA was used to print biodegradable polymers, ceramic acrylate, or hydroxyapatite for bone reconstruction [22,23,24]. Lithography-based ceramics manufacturing (LCM) was employed for the high precision fabrication of glass ceramic dental replacements [25]. Another 3D printing technology, the digital light processing (DLP) based on photopolymerization, was employed for the fabrication of zirconia implants [26,27]. In selective laser sintering (SLS), a powder fusion printing (PFP) technique, granules of metal, raisin or plastic are beamed with a laser to fuse in a layer-after-layer fashion [28]. Tricalcium phosphate and hydroxyapatite were used with this technique to produce scaffolds for bone regeneration [29]. The advantage of the PFP techniques is the possibility to print melting metals such as titanium, magnesium or cobalt chromium, employed in medicine and dentistry. Solid form fabrication (SFF) allows deposition of strands by a nozzle via a precise XYZ axes positioning system. Upon extrusion, however, the material must retain its shape. As an example, polycaprolactone (PCL) was combined with alginate to print scaffolds for cartilage repair [30].

The 3D printing technology that includes cells has been named “bioprinting”, and the hydrogels, in which cells reside for the printing purpose, have been named “bioinks” [31]. Hydrogels offer modifiable chemical composition, and adjustable mechanical and biodegradation properties [32]. Hydrogels represent attractive materials for bioinks due to their biocompatibility, low cytotoxicity, and high water content [33,34]. A hydrogel suitable for 3D bioprinting must be viscous enough to keep its shape during printing, without squeezing cells, and have cross-linking abilities to allow retention of the 3D structure after printing. In extrusion bioprinting, pneumatic (pressure) or mechanical (plunger) force extrudes filaments. Fast gelation for retention of the desired outer form and inner structure ensues. As examples, alginate is combined with calcium, and fibrinogen with thrombin. The main advantage of extrusion bioprinting is the possibility to use multiple materials and cell types in different combinations [5]. Laser-assisted bioprinting (LAB) is based on a laser pulse that produces local heating of a cell-containing solution causing dropping of cells in an orderly manner on the other side of a platform/substrate [35]. Laser-direct-writing, a type of LAB, was successfully used to deposit different cells types and biomaterials [36]. In inkjet bioprinting, a defined volume of fluid (with or without cells) is jetted onto a platform to obtain a precise pattern [5]. Droplets are deposited using either thermal or piezoelectric energy. The major advantage is the speed achieved in building the complex cell-laden tissue mimicking equivalents, and a multi-head approach for bioprinting different cell types and biomaterials. The main disadvantage is that cells or bioactive molecules must be in a liquid state to allow deposition, and subsequently solidify into the required structure. The commonly used hydrogels in LAB techniques are cross-linked using physical, chemical, pH, or ultraviolet light methods [37]. A comprehensive comparison of both types of 3D printing techniques relevant for tissue constructs has been recently published [3].

## 3. 3D Printing for Tissue Engineering

3D printing has been very successful in making biomaterial scaffolds with custom-designed geometries and is becoming an important technology for tissue engineering [38]. The tissue engineering approach aims at rebuilding a functional tissue that could either replace or facilitate the regeneration of the missing tissue [39]. The tissue engineering triangle comprises biomimetic scaffolds as the initial structural support, cells as tissue masons and bioactive molecules as the instructors providing the necessary signals [40]. In the past, the production of a tissue relied on scaffold fabrication techniques with limited possibilities to reproduce the tissue complexity. Today, the 3D printing approach has a distinct advantage in that it can produce various geometries to perfectly fit any tissue defect as well as mimic complex inner tissue architecture and heterogeneity via the precise positioning of different materials and/or cell types [1,2]. For hard tissues, 3D printing of bone graft scaffolds comprised approaches using natural and synthetic biomaterials [41,42] assembled in a biomimetic scaffold [43]. For soft tissues, 3D printing mainly relied on various hydrogels combined with cells to produce tissues like cartilage [44,45,46,47,48], vascular as well as cardiovascular tissues [49,50,51], liver [52], and skin [53,54,55,56,57,58,59]. Recently, modular assembly, with separate 3D-printed biological components (cells, cell aggregates or microtissue units) combined with the corresponding biomimicking scaffolds, has been applied for 3D printing of blood vessels, osteochondral grafts or liver constructs [60]. Companies have also exploited 3D printing to biofabricate different types of tissues. exVive3D™ Liver (Organovo, San Diego, CA, USA) is a bioprinted human hepatic tissue successfully used for toxicity assessment to complement in vitro and preclinical testing [61]. TeVido (TeVido Biodevices, Austin, TX, USA) is developing breast reconstructions for cancer patients based on their own cells, and l’Oréal (Paris, France) and Poietis (Pessac, France) work together to tackle hair loss by 3D printing hair follicles [62]. A recent detailed and comprehensive description of different 3D printing methods with their advantages and disadvantages, clinical applications, the necessary biomaterial considerations and bioprinting strategies provides an excellent guidance for the biofabrication of tissue constructs [63].

## 4. 3D Printing in Dentistry: A Brief Overview

**Figure 1:**
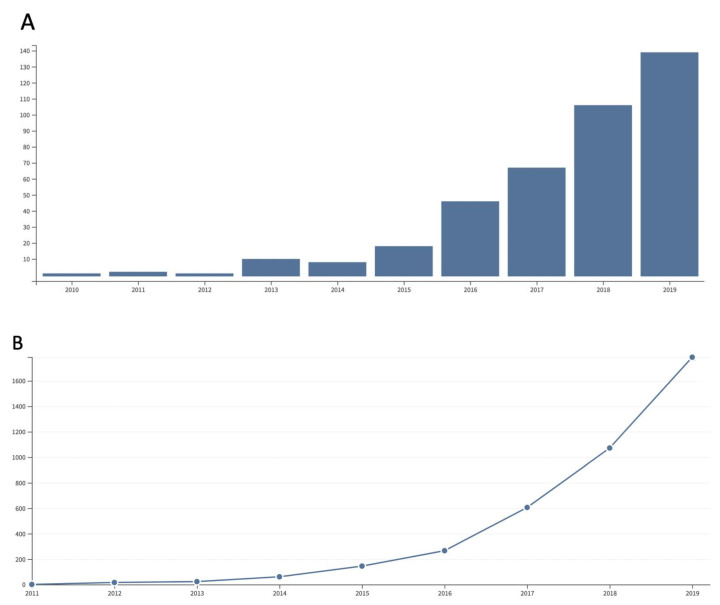
A notable increase in the number of articles (**A**) and citations (**B**) published on 3D printing in the dental field during the last decade. Source: Web of Science.

3D printing in the dental field was introduced more than a decade ago and its application continues to increase, with 139 publications and 1800 citations in 2019 (Figure 1). SLA manufacturing of implant-drill-guides for guided surgery procedures and laser–sintered alloys were the first additive fabrication technologies applied in dentistry. The development of digital image acquisition, and the application of the CAD/CAM technology allowed the emergence of a fully digitalized dental treatment [64]. Intraoral-scanning has been replacing plastic imprints to produce computer-aided manufactured (CAM) digital physical models. Hence, the manual handling is being replaced throughout the three processing steps, and this novel approach has been termed “digital workflow” [65]. The first step comprises data acquisition through various scanning technologies. The most common techniques are computerized tomography (CT), cone beam computed tomography (CBCT), magnetic resonance imaging (MRI), and laser digitizing with extraoral or intraoral scanning devices. The second step is the data processing and the model design with a computer-aided design (CAD) software. The resulting STL file is imported into the printer software. The building variables and parameters for segmentation are next specified, together with the support structures, to generate the information needed to run the 3D printer. In the third step, the processed data are used to manufacture structures with the chosen material through the CAM step [65]. 3D-printed objects have been successfully used in prosthodontics, orthodontics, orthognathics, endodontics, craniofacial, and oral and maxillofacial surgical procedures [66]. The benefits include simplification, minimal invasiveness, greater accuracy, a reduction in operating times, and improvement in patient comfort and aesthetics.

### 4.1. Presurgical Virtual Planning and Dental Surgical Guides

Haptics technology exploits the sense of touch and its interaction with the virtual environment. The convergence of haptics and virtual reality technology and integration with 3D imaging data resulted in the emergence of dental haptic simulators. Created virtual oral anatomy and facilitated the simulation of dental procedures offer real-time visual, tactile and auditory planning as well as feedback [67,68]. Combination of haptic instruments with 3D printing contributed towards the development of patient-specific instrumentation, in particular, surgical guide instruments that increase accuracy during surgery while decreasing the risk of infections and operation time/cost.

Customized design of surgical splints and stainless-steel arch-wires through 3D digital treatment simulation allows for precise fabrication as well as the prediction of dental and jaw movements. This approach reduces treatment time, reinforces decompensatory tooth movements, and rapidly improves aesthetics [69]. Several commercial applications have been developed to facilitate 3D virtual treatment planning, although the biomechanical planning of tooth movements requires further development. Surgical planning software including Virtual Surgical Planning (VSP^®^) Technology (3D Systems; Littleton, CO, USA), ProPlan CMF™ (Materialise, Leuven, Belgium), IPS CaseDesigner^®^ (KLS MÂRTIN Group, Tuttlingen, Germany), and InVivo6^®^ (Anatomage, San Jose, CA, USA) integrate CT/Cone Beam CT (CBCT) data, 3D stereophotogrammetry, and intra-oral occlusal scans to generate a comprehensive 3D model. Dental movements and surgical osteotomies can be simulated interactively between the surgeon, orthodontist, and engineer. The final clinical plan is used to generate an intermediate and a final splint, both of which are fabricated via 3D printing. Virtual orthodontic movements can be similarly planned and applied. Software such as InVivo^6®^ (Anatomage, San Jose, CA, USA) and Orchestrate^®^ (Orchestrate3D, Rialto, CA, USA) incorporate data either from CBCT or intraoral scans and allow for individual tooth movements to be programmed and sequenced. The orthodontist creates a virtual set-up of the final occlusion, as well as the sequence and the pathway for tooth movements. Sequenced models or aligners can be fabricated with a relatively inexpensive 3D printer in a dental laboratory or in the orthodontist’s office. Similar approaches using fixed appliances were developed by SureSmile^®^ (OraMetrix; Richardson, TX, USA) and Insignia^®^ (Ormco, Orange County, CA, USA) to fabricate custom arch-wires or orthodontic brackets. The possibility to determine the precise sequence of tooth movements results in their perfect alignment.

3D printing has been used to produce surgical guides for pulp canal obliteration based on CBCT scans. To diminish the risk of perforation by producing a correct path of canal and instrumentation access, guides were printed and utilized to target burs to otherwise elusive canal spaces [70]. 3D printing was also used to print a replica of a tooth to be autotransplanted, in order to prepare the implantation site and decrease PDL damage from repeated insertion/removal cycles during fitting [71].

### 4.2. Educational Models in Dentistry

In academia, in the past, dental students had to rely on extracted teeth, human cadavers, resin blocks or commercially prepared teeth replicas for the simulation of cases during their studies [72,73]. In clinics, printed tooth reproductions were used in preparation for the treatment of complicated cases to simulate optimal access, instrumentation and obturation [74]. Today, 3D-printed objects represent a teaching aid for students to improve their understanding of the complexity of different oral structures, to simulate functions and to train for the optimal intervention. Duplicate 3D-printed models are used for standardized students’ skill assessments as well as individual student skill progression.

In dental practice, 3D-printed models could improve communication between the practitioner and the patient. Better understanding of the proposed treatment leads to a compliant attitude and develops mutual understanding and trust [75].

In research, a three-dimensional organ-germ culture method that generated a structurally correct tooth [76,77] was replaced by a 3D-printed bioengineered tooth replica for in vitro and in vivo experiments toward understanding the whole-tooth morphogenesis [78] as well as regeneration [78,79].

### 4.3. 3D Printing for Reconstruction of Oral Tissues

The periodontal ligament (PDL) is the fibrous connective tissue structure that anchors alveolar bone to tooth cement [80]. By resisting compressive loading, PDL allows tooth movement upon mastication and speech. During the initial inflammatory processes and subsequent periodontium wound healing, the blood supply through the PDL vascular plexus and the neural network play critical roles [79,81]. Hence, the loss of PDL impairs not only teeth physiological movement but also the defense against infection [82]. PDL-derived cells possess mesenchymal stem cell-like properties and have been considered as a source for the reconstruction of periodontal tissues [83,84]. More than two decades ago, PDL-derived cells were used with the ‘‘cell sheet technology’’, i.e., cell detachment without enzymatic treatment [85] for periodontal regeneration. Preclinical and clinical studies demonstrated periodontal regeneration with inserted PDL fibers and newly formed cementum in periodontal defects [86,87,88,89,90,91]. The major drawback of the cell-sheet approach was the compromised biomechanical stability and the demanding surgical technique. The improvements of the cell sheets’ biomechanical properties included layering of several sheets, supporting the sheets with hydrogels, and adding ECM components to the thermo-responsive surface [90,92]. With the development of additive manufacturing, a 3D-printed calcium phosphate (CaP)-coated PCL scaffold was combined with cell sheets from different human cell types resulting in significant periodontal attachment [93]. In another approach, decellularized periodontal ligament cell sheets were transferred onto melt electrospun PCL membranes. The retained intact extracellular matrix and resident growth factors supported repopulation by allogeneic cells [94]. A recent study demonstrated the formation of a periodontal-like structure around a titanium implant. PDL cell sheets were cultured on an acid-etched, blasted titanium surface coated with calcium phosphate to mimic the environment around a natural tooth [95].

The 3D printing approach could prove particularly valuable in answering the need for the complex hierarchical organization of periodontium consisting of gingiva, PDL, cementum, and alveolar bone. The periodontium is a highly organized tissue that supports the teeth and plays an important role in transmitting mechanical forces [80,96]. Reconstruction of periodontal tissue necessitates coordinated spatiotemporal control of the healing process via volume maintenance, wound stabilization and selective cell repopulation [97]. The approach with multiphasic biomaterial constructs could recapitulate the structural integrity of tooth-supporting tissues destroyed as a consequence of trauma, chronic infection or surgical resection. A series of consecutive studies aimed at developing 3D-printed biomimetic composite hybrid polymeric scaffolds to reproduce the dentin–PDL–bone interfaces [98,99,100]. The studies relied on a differential structural design for the alveolar bone and PDL parts, using 3D printing with PCL for bone and PGA for PDL, genetically modified human cells and human tooth dentin slice [98]. The newly formed tissues consisted of parallel and obliquely oriented fibers that grew within the PCL/PGA constructs forming tooth cementum-like tissue, ligament, and bone structures. In the next study, PCL was combined with human cells for producing PDL and bone structures and evaluated in an in situ rat mandible defect model [99]. The design of perpendicularly oriented micro-channels of the PDL part allowed the formation of oriented anchoring ligaments linking cement and alveolar bone [99,100]. The “guided” fiber PDL architecture permitted control of tissue infiltration and optimal organization of both ligament interfaces. This knowledge was subsequently applied for the treatment of the periodontal reconstruction case following the “digital workflow” approach [101]. After the CBCT scan of the defect area, an STL file was created and used to design the osseous defect together with guided PDL channels. PCL was combined with hydroxyapatite and 3D printed. The construct was additionally submerged in bb-PDGF. The treated site remained intact for one year, after which the construct presented problems and had to be removed. Further research on the refinement of the guided “pillars” for PDL identified combined mesoscale and microscale hierarchical features allowing cell alignment for a more precise PDL formation [102]. An approach from another group consisted of a 3D-printed triphasic PCL/hydroxyapatite scaffold corresponding to cementum, PDL, and alveolar bone, each loaded with the three corresponding cell types and timely delivery of growth factors [103]. In vivo implantation resulted in aligned PDL-like collagen fibers that inserted into bone-like and dentin/cementum tissues. This approach illustrates a strategy for the regeneration of multiphase periodontal tissues by spatiotemporal delivery of several cell types and signaling proteins. Together, these studies demonstrate the potential of 3D printing to generate customized periodontal scaffolds for the regeneration of multi-tissue interfaces required for oral, dental and even craniofacial engineering applications.

## 5. Biomaterials Used for 3D Printing of Oral Tissues

Scaffolds produced from biomaterials provide an initial mechanical support and allow for cell population, adhesion and differentiation to foster guided tissue regeneration. The majority of the raw materials for additive manufacturing used for dental and medical purposes can be grouped into binder/powder material combinations including polymers (resins and thermoplastics), ceramics, and metals [104]. Biomaterials for tissue fabrication can be broadly divided into inorganic, mainly used for bone regeneration and organic, predominantly used for soft tissue regeneration. Inorganic biomaterials need to be mechanically stable, resorb slowly, and not induce an inflammatory reaction [105]. Hydroxyapatite is stoichiometrically similar to the mineral phase of the natural bone ensuring biocompatibility yet has reduced mechanical resistance and a long resorption time. Calcium phosphate binds chemically to bone, it is easier to manufacture into desired shapes and resorbs faster compared to hydroxyapatite [106]. In contrast to hydroxyapatite and calcium phosphate, the production of bioglass allows for an extremely versatile composition leading to a controlled resorption rate and modulation of cell migration and tissue revascularization [107]. Organic biomaterials are polymers of natural origin such as agarose, alginate, collagen, gelatin, chitosan, fibrin, or synthetic such as polylactide (PLA), poly glycolic acid (PGA), poly-lactic-*co*-glycolic acid (PLGA), and polycaprolactone (PCL) [106]. Hydrogels used for soft tissue regeneration can be either curable polymers, producing mechanically solid scaffolds upon solidification, or soft, injectable hydrogels. Both can be combined with cells; in the first case, cells are seeded after curing to avoid harsh printing/curing conditions; in the second, cells reside within the bioink during printing (bioprinting). A hybrid barrier membrane has been recently produced for guided tissue regeneration by 3D printing by combining gelatin (for cell adhesion), elastin (for membrane long-term stability and elasticity) and sodium hyaluronate (for cell-signaling), and cross-linked by 1-Ethyl-3-(3-dimethylaminopropyl) carbodiimide (EDC) [108]. The membrane has small pores on one side and large pores on the other side to accommodate osteoblasts, fibroblasts, and keratinocytes population on the different sides. The in vitro analysis indicated biocompatibility, mechanical strength, degradation rates, as well as tensile modulus for easy surgical handling.

Hydrogels are capable of absorbing and retaining large quantities of water. They can be classified into naturally-derived hydrogels such as agarose, alginate, fibrin, collagen type I, chitosan, gelatin, hyaluronic acid, Matrigel^TM^, and synthetically-derived and synthetically-derived such as Pluronic^®^-127, polyethylene glycol (PEG) or various methacrylated combinations including gelatin (GelMA), hyaluronic acid (HAMA), silk fibroin (SilMA), and pectin (PECMA) [1,106,109,110]. The bioprintability of hydrogels is governed by their rheological properties and the target bioprinting modality, and includes three bioprinting techniques: extrusion-based, droplet-based and laser-based (cell transfer or photopolymerization) [110]. Two printing approaches: extrusion-based bioprinting for cell-encapsulating hydrogels and melt electro-writing for aligned sub-micrometer fibers were converged to produce a mechanically stable construct with viable cells [111]. Bioink gelation can be achieved via physical (temperature, ions), chemical (glutaraldehyde, genipin, irradiation-induced photo-polymerization) or enzymatic (thrombin) crosslinking. Due to hydrogels’ high permeability to oxygen, nutrients and other water-soluble compounds, they are considered as attractive materials for the fabrication of tissue constructs. Another important advantage of the 3D printing approach with hydrogels is the easy incorporation of bioactive agents [112]. The presence of such signaling molecules can provide the necessary instructions to residing, host-tissue cells or externally delivered cells for facilitated tissue regeneration. Bioinks were also produced from decellularized matrix components, cellulose or silk [31]. Bioinks derived from decellularized extracellular matrices present major advantages: they contain all tissue components preserved in the correct proportions, and the tissue-specific signaling factors, therefore providing an optimal instructive environment for cell migration, proliferation and differentiation [113]. Such bioinks have been successfully bioprinted into porcine liver, heart, skin, cartilage and skeletal muscle tissues, and human adipose tissue [114,115].

## 6. Oral Soft Tissue Regeneration: Current Treatments and Limitations

Oral soft tissue plays an important role in the structure and function of the oral cavity. The oral mucosa covers the inside of the oral cavity and consists of: (1) the masticatory mucosa (gingiva and cover of the hard palate), (2) the specialized mucosa (cover of the tongue), and (3) lining mucosa [80]. Gingiva belongs to masticatory mucosa, covering the alveolar bone and surrounding the teeth. Structurally, it consists of the oral epithelium and the underlying connective tissue, lamina propria. The non-attached alveolar mucosa consists of a thin, non-keratinized stratified squamous epithelium and loosely connected collagen fibers. In contrast, the attached mucosa contains the thick, keratinized squamous epithelium and well-organized and dense collagen fibers. The hard palate and attached gingiva are made of the keratinized type of attached mucosa. The attached keratinized mucosa is indispensable for the maintenance of teeth, PDL, as well as dental implants. It forms a protective barrier against harmful environmental agents such as pathogens, chemicals, and constant abrasion [116]. The insufficiency of oral mucosa due to gingival recessions, infections, trauma, and tumors require oral mucosa reconstruction. Soft tissue augmentation is frequently used to regain reduced or lost tissue in edentulous patients, cover an exposed root or implant, increase buccal mucosal soft tissue thickness or coronal soft tissue height [117,118]. The treatment of choice must comply with functional mastication, speech, and aesthetics. Depending on the location and the need, various techniques are used, most relying on the autologous tissue grafts. For the soft tissue volume augmentation, subepithelial connective tissue graft (SCTG) gave a better clinical outcome compared to free gingival grafts (FGG), and it is used at implant sites or in partially edentulous patients [117,119]. However, the use of an autologous tissue graft presents several disadvantages and limitations: the height, length, and thickness of the palate depends on the anatomical position and varies among patients; the harvesting technique is surgically demanding, a limited amount of tissue can be gained per intervention, and patients complain about prolonged postsurgical pain and numbness [120,121,122,123,124]. To reduce the morbidity caused by graft harvesting, soft tissue substitutes have been sought [125,126]. The requirements for an ideal non-autologous graft for soft tissue augmentation comprise biocompatibility, volume and mechanical stability, concomitant biodegradability and tissue integration, easy handling, and low cost without compromised efficacy [126]. Freeze-dried skin allografts were among the first products introduced in mucogingival surgery. They were initially used as a replacement for FGG in combination with an apically positioned flap for the augmentation of keratinized tissue [127]. Later in the 1980s, allogenic dermal substitutes such as the acellular dermal matrix graft, Alloderm^®^, (Life Cell Corporation, The Woodlands, TX, USA), originally developed for covering full-thickness burn wounds [128], were introduced to increase keratinized tissue, cover exposed roots, deepen the vestibular fornix, and augment localized alveolar defects [129,130,131,132]. Unfortunately, the outcomes were associated with difficult clinical handling and high shrinkage rates of the grafted areas. Moreover, histology analysis indicated a significant difference in comparison to the natural tissue [133]. To reduce scar retraction and enhance the healing process, a novel collagen matrix, Geistlich Mucograft^®^ (Geistlich Pharma AG, Wolhusen, Switzerland), was designed and evaluated as a replacement for autogenous tissue to increase the width of keratinized tissue and cover gingival recessions [134,135,136,137,138]. Clinical data indicated strong enhancement of the keratinized tissue width with similar outcomes in comparison to the FGG [139,140,141,142]. Another matrix, Mucoderm^®^ (Botiss Dental, Berlin, Germany), a porcine dermis-derived acellular matrix, was used for the treatment of oral dehiscence, ridge preservation, root coverage and vertical augmentation [143]. Finally, a highly porous yet volume stable 3D matrix consisting of slightly cross-linked reconstituted collagen fibers has been introduced (Geistlich Fibro-Gide^®^, Geistlich Pharma AG, Wolhusen, Switzerland) and shown to increase soft tissue volume similarly to SCTG [144,145,146]. These promising biological scaffolds reduce morbidity, decrease surgical time as well as costs. However, they must be tailored for each individual defect, do not reproduce the inner architecture of a particular oral site, and remain surgically demanding.

## 7. Platelet Rich Fibrin (PRF) for Oral Soft Tissue Regeneration

The first steps during the wound healing process, including oral soft tissue, are haemostasis and formation of granulation tissue, both orchestrated by the signaling molecules released by various cell types. To accelerate the healing process at a surgery site, blood concentrates rich in platelets and autologous growth factors have been developed [147,148]. The first blood concentrate, platelet rich plasma (PRP), was obtained after platelets separation from red blood cells during a centrifugation process [147,149]. This preparation required an anticoagulant and relied on thrombin for subsequent clotting. The alternative concentrate, platelet rich fibrin (PRF), was obtained without anti-coagulants, with clotting taking place gradually and naturally [150]. While fast coagulation (PRP) results in a quick release of growth factors and dense fibers formation, slow coagulation (PRF) leads to long-term growth factors release from a more compact matrix rich in fibers [151,152]. Both blood preparations, PRP and PRF, have been extensively studied for a plethora of clinical problems [153]. Over the years, PRF has gained more interest, as it is less time consuming, does not require an anti-coagulant or thrombin, and due to the preserved fibrin matrix ultimately favors neovascularization. Several improvements have been made to the initial PRF preparation to increase cells and matrix longevity. The centrifugation speed was decreased resulting in an increase in the number of platelets and leukocytes, and a more balanced distribution of cells within the matrix [154]. An additional decrease in the centrifugation time further improved cell survival and growth factor release [155]. This low-speed centrifugation concept was also applied for the liquid injectable PRF, with similar results: selective enrichment of platelets, growth factors and leukocytes [156,157].

For soft tissue augmentation in dentistry, PRF was mainly employed for the treatment of extraction sockets, gingival recessions, and palatal wound closure [158]. Although beneficial effects were seen, conclusions were difficult to draw due to the lack of proper controls in study designs. A recent review analyzed studies that used PRF for different dental treatments, namely in endodontics, implantology, sinus lift, socket preservation, bone regeneration, and socket preservation, orthodontics and periodontology [159]. In periodontology, PRF was often combined with biomaterials and demonstrated beneficial outcome. The authors hypothesize that PRF made the acellular matrix more cell-friendly, fostering better adhesion, cell–cell communication, and tissue integration. A similar role for PRF can be envisioned for the 3D-printed, individualized acellular scaffolds. However, the main limitation remains the lack of standardized protocol of PRF preparations among clinicians.

## 8. Monitoring Soft Tissue Augmentation

For an accurate and standardized assessment of the soft tissue augmentation requirements and the subsequent different treatment outcomes, measurement of the surface and thickness, i.e., volume of the soft tissue, is crucial. A recent review addressed technological developments from 2D to 3D methods outlining advantages and drawbacks [160]. Traditional, 2D methods for measurement of soft tissue comprise a periodontal probe, oral photography and ultrasonic devices. Their main advantages are their relative non-invasiveness and accuracy of 0.1–0.5 mm. The significant limitation for all three methods is the need for a connection to a 3D design software in order to obtain 3D information of the defect areas. 3D methods comprise CBCT, Moiré method and laser CAD/CAM devices. CBCT is limited due to linear measurements, scattering effect, limited accuracy and radiation exposure, but it is painless. The Moiré method is time-consuming, requires casting with risks of displacement and dimensional changes during impression, but provides more accuracy compared to CBCT. Lasers were proven as the most accurate and offer a choice between scanning of an imprinted cast and a direct oral digital scanning. Digital optical scanning and assessment methods have been introduced to measure and longitudinally quantify soft tissue volume loss or gain [161,162]. Therefore, the “digital workflow” can also be employed for the initial assessment (diagnostic), virtual planning and evaluation of the efficacy of treatment options and future 3D printing of soft tissues required for gingival soft tissue augmentation.

## 9. Tissue Engineering for Oral Soft Tissue Regeneration

Tissue engineering approaches have already been developed with the aim to establish 3D organotypic cultures resembling the natural gingiva for clinical as well as research purposes. An ideal full-thickness tissue engineered gingiva should consist of: (1) a supporting connective tissue, i.e., lamina propria containing fibroblasts within a vascularized ECM; (2) a continuous basement membrane which separates lamina propria from the epithelium, and (3) a stratified squamous epithelium containing densely packed keratinocytes that undergo differentiation as they move towards the surface. Initially, keratinocytes were cultured in cell sheets with a cell feeder layer [163], resulting in a fragile, difficult to handle and retractable tissue. Subsequently, incorporation of fibroblasts and collagen provided the support of a lamina propria substitute and led to the fabrication of the first gingiva equivalent tested in clinics [164]. Scaffolds that were developed and used in gingiva tissue engineering in the past decades can be classified into: (1) naturally derived (acellular human dermis), (2) collagen-based, (3) fibrin-based, (4) gelatin-based, (5) synthetic (PCL) or hybrid [165]. For clinical applications, the primary cell source was cells isolated from autologous biopsies in contrast to the in vitro studies which often favored immortalized cell lines for the sake of availability, reproducibility, and standardization. However, cancer-derived cell lines regularly present compromised physiological responses. Keratinocytes and fibroblasts were therefore “physiologically” immortalized by the expression of Telomerase Reverse Transcriptase [166]. These cells allowed the formation of a full-thickness gingival equivalent that closely reproduced the native gingival tissue architecture [167]. Such organotypic models provide invaluable tools to study oral mucosa biology and could also replace animal studies for drug targeting, vaccination development, and testing of new therapeutics. In laboratories, they are used to understand the physiological role of human oral mucosa barrier properties as well as different pathologies, including oral cancer, bacterial and fungal infections. Additionally, oral mucosa models are used for cytotoxicity and biocompatibility testing of oral health care products [168]. In clinics, tissue engineered gingiva was used to augment keratinized tissue around teeth [169] and has recently been up-scaled for large (over 15 cm^2^) soft tissue defects [170]. Gingival grafts cultured on a biodegradable collagen scaffold were also employed in periodontal plastic surgery to treat patients with insufficiency of the attached gingiva [171,172].

Several companies have ventured into developing gingival tissue models. SkinEthic Laboratories (Nice, France) offers an epithelial gingival model based on the air–liquid interface culture of normal gingival keratinocytes. This keratinized, stratified, squamous epithelium can be used as a screening tool for corrosion, irritation, permeability and metabolism testing of new compounds as well as for investigating the effects of anti-inflammatory or antibiotic formulations [173]. MatTek Corporation developed EpiOral™, a model of human oral (buccal) stratified non-keratinized epithelium, and EpiGingival™, a model of gingival stratified keratinized epithelium for screening newly developed oral care products as well as for studying innate immunity, drug delivery, and pathology of the oral mucosa.

## 10. The Future: 3D Printing for Oral Soft Tissue Regeneration

**Figure 2:**
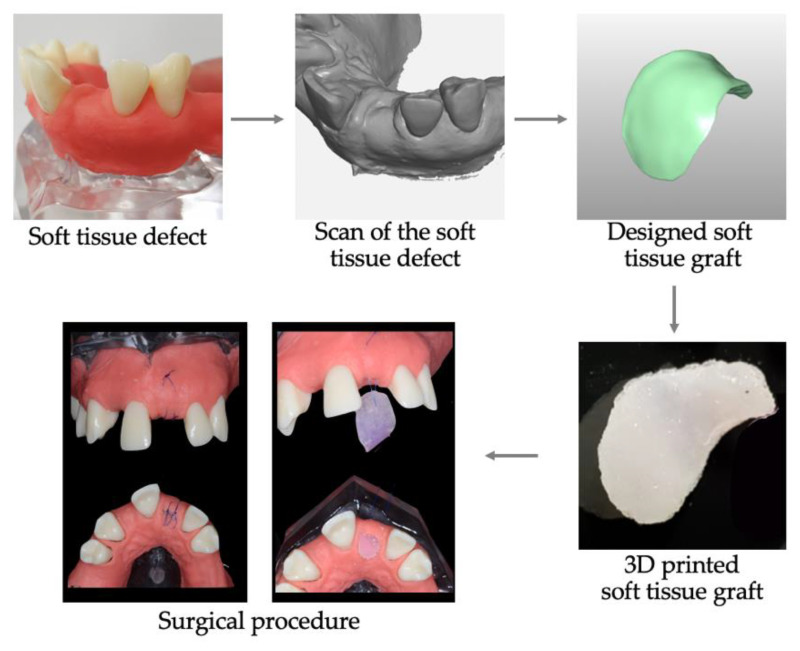
“Digital workflow” for soft tissue augmentation. The soft tissue defect is scanned (intraorally or from the imprint-derived cast); the ideal graft is designed and converted into an STL file. Upon 3D printing of a defect-tailored graft for optimal volume augmentation, the graft is surgically placed to fit the defect, and sutured.

3D printing could prove an ideal approach to produce scaffolds for soft tissue augmentation by addressing the variability in the soft tissue shape, inner architecture, thickness, volume, mechanics, and function associated with the position in the oral cavity. Importantly, 3D printing would allow application of the “digital workflow”, resulting in the production of the patient-tailored grafts. Several decisions would need to be made to establish the 3D printing approach of oral mucosa [6]: the most appropriate imaging acquisition, the choice of biomaterial to best correspond to gingiva in its chemical, biological and mechanical properties, inclusion or not of cells (and the source), and finally the choice of the printing technique. Digital imaging of bone, soft tissue, and blood vessels during pre-operative virtual planning for face reconstruction has been accomplished with Haptics system [67]. With the intraoral scan digital acquisition, the level and the anatomy of tissue insufficiency, as well as the vascular network, can be determined. The desired characteristics of 3D printable biomaterials comprise biocompatibility, high porosity to promote cell population, tissue in-growth and vessel formation, biodegradability according to the rate of new matrix deposition (tissue generation), and mechanical stability. The appropriate macro-architecture characteristics would ensure timely neovascularization, as recently demonstrated for the regeneration of dental pulp [174]. A smart biomaterial containing all instruction cues could circumvent the need for growth factors or cells. However, in certain pathological cases such as inflammation, infection or necrosis, different anti-inflammatory, and immunomodulatory drugs or antibiotics could be incorporated and released in a timely and concentration-controlled manner. The inclusion of approved autologous blood concentrate preparations, such as PRF or PRP, could facilitate the healing process via the release of natural growth factors. From the dentist’s point of view, the “digital workflow” would have to be easy to plan and execute, with the final soft tissue graft that is effortless to handle and suture and provides satisfactory functional as well as esthetical results. A schematic illustration of the potential future “digital workflow” is depicted in Figure 2.

In summary, 3D printing is a versatile manufacturing technology offering vast patterning possibilities, precise manufacturing, and abundant choices of biomaterials for a cost-effective patient-tailored end construct. This interdisciplinary approach pursues the integration of technologies from the fields of engineering, digital imaging, materials science, biology, chemistry, and medicine. 3D printing technology has already been largely employed in numerous biomedical applications to make tissues, organs, and medical devices, as well as to provide surgical planning aids and educational models. Continuous expansion and adaptation of 3D printers’ abilities, combined with reduced costs, increased speed, and use of a broader range of printable materials will bring this technology to the forefront of biomedical applications. New challenges, needs, and achievements can be envisioned in the field of bioprinting as more researchers with different backgrounds and research questions employ 3D printers. In dentistry, particularly for soft tissue regeneration, application of the “digital workflow” to achieve a perfect-fit patient-tailored graft according to the defect, with an adjusted inner architecture and outer shape to maximize tissue mimicry, will result in functional as well as aesthetically pleasing tissue restoration.

## Figures and Tables

**Table 1:** A timeline depicting the evolution of the three-dimensional (3D) printing technologies of importance for the medical field.

Year	Key Developments
1984	Invention of stereolithography (SLA) 3D printing (Charles Hull)
1986	Invention of the selective laser sintering (SLS) process (Carl Deckard)
1988	Bioprinting by 2D micro-positioning of cells and the first commercial SLA 3D printer (Charles Hull)
1989	Patenting of a fused deposition modelling (Lisa and Scott Crump)
1999	First 3D-printed organ—a bladder—used for transplantation (Wake Forest Institute for Regenerative Medicine)
2000	EnvisionTEC launched the first commercial extrusion-based bioprinter, the 3D-Bioplotter
2002	First early stage kidney prototype bioprinted via microextrusion (Wake Forest Institute for Regenerative Medicine)
2003	First inkjet bioprinter (modified HP standard inkjet printer)
2005	Founding of RepRap, an open source initiative to build a 3D printer that can print most of its own components
2007	Selective laser sintering printer becomes available, for 3D parts fabrication from fused metal/plastic
2008	First 3D-printed prosthetic leg
2009	First 3D-printed blood vessels (Organovo)
2012	First 3D-printed jaw
2014	First 3D-printed human liver tissue (Organovo), and first desk-top bioprinter (Allevi)
2015	First implanted 3D-printed bioresorbable scaffold for periodontal repair (University of Michigan)
2018	First commercial 3D-printed full human tissue (skin) model Poieskin (Poietis)
2019	First 3D-printed heart that contracts, with blood vessels (University of Tel Aviv) and 3D-printed lung air-sac with surrounding blood vessels (Volumetric)
2020	3D printer for personalized medicine M3DIMAKER (FabRx)

Adapted from GlobalData, “The history of 3D printing”, Carlos Gonzales, ASME, and [3].
